# Is trade liberalisation a vector for the spread of sugar-sweetened beverages? A cross-national longitudinal analysis of 44 low- and middle-income countries

**DOI:** 10.1016/j.socscimed.2016.11.001

**Published:** 2017-01

**Authors:** Ana Mendez Lopez, Rachel Loopstra, Martin McKee, David Stuckler

**Affiliations:** a Department of Sociology, University of Oxford, OX1 3UQ, United Kingdom; b ECOHOST, London School of Hygiene & Tropical Medicine, WC1H 9SH, United Kingdom

**Keywords:** Low-and middle-income countries, Trade liberalisation, Tariffs, Sugar-sweetened beverages

## Abstract

Does trade and investment liberalisation increase the growth in sales of sugar-sweetened beverages (SSBs)? Here, for the first time to our knowledge, we test this hypothesis using a unique data source on SSB-specific trade flows. We test whether lower tariffs effectively increase imports of SSBs, and whether a higher level of imports increase sales of SSBs. Cross-national fixed effects models were used to evaluate the association between SSBs sales and trade liberalisation. SSBs per capita sales data were taken from EuroMonitor, covering 44 low- and middle-income countries from 2001 to 2014, SSBs import data were from TradeMap, Foreign Direct Investment data were from EuroMonitor, and data on applied tariffs on SSB from the World Trade Organisation tariffs database, all 2015 editions. The results show that higher tariffs on SSBs significantly decreased per capita SSB imports. Each one percent increase in tariffs was associated with a 2.9% (95% CI: 0.9%–5%) decrease in imports of SSBs. In turn, increased imports of SSBs were significantly associated with greater sales of SSBs per capita, with each 10 percent increase in imports (in US$) associated with a rise in sales of 0.36 L per person (95% CI: 0.08–0.68). Between 2001 and 2014, this amounted to 9.1 L greater sales per capita, about 40% of the overall rise seen in this period in LMICs. We observed that tariffs were inversely but not significantly associated with sales of SSBs. In conclusion, lower tariffs substantially increased imports of SSBs in LMICs, which translated into greater sales. These findings suggest that trade policies which lower tariff barriers to SSB imports can be expected to lead to increased imports and then increased sales of SSBs in LMICs, with adverse consequences for obesity and the diseases that result from it.

## Highlights

• Higher tariffs on SSBs significantly decreased per capita SSBs imports in LMICs.
• Increased imports of SSBs were significantly associated with greater sales of SSBs.
• Policies that liberalise trade of SSBs can be expected to lead to the spread SSBs.

## 1 Background

**Fig. 1:**
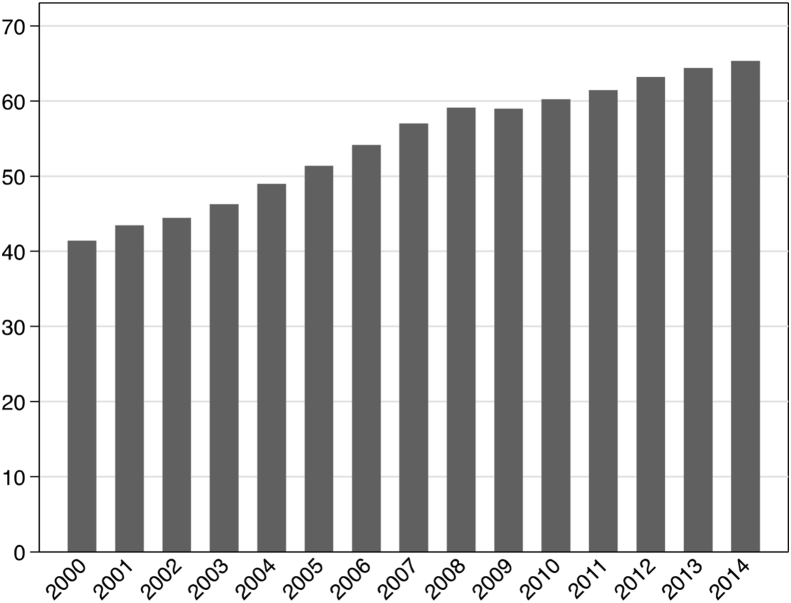
Average per capita sales of SSBs litres for 44 LMICs, 2000–2014.

Since 2001, there has been substantial increase in sales of sugar-sweetened beverages (SSBs) drinks in low- and middle-income countries (LMICs) (Popkin and Hawkes, 2015, Stuckler et al., 2012) (Fig. 1). Sales grew at 3.9% per annum during this period, rising from 43.4 L per capita in the year 2001 to 65.3 L per capita in 2014, an overall increase of 50.1%. In some regions, such as Latin America, annual per capita sales now exceed 100 L per capita per year (Euromonitor International, 2015).

The link between consumption of SSBs and disease is now established, with increased risks of obesity, tooth decay, and diet-related non-communicable diseases (NCDs) (Basu et al., 2013a, Basu et al., 2013b, De Koning et al., 2012, Imamura et al., 2015, Monteiro, 2009). Based on two systematic reviews showing a link between a higher intake of free sugars (added sugars and juice sugars) and higher rates of overweight and dental caries, in 2015 the World Health Organisation (WHO) recommended reducing intake of free sugars to less than 10% of total energy across the life course, and suggested that intake might be reduced further, to below 5% of total energy intake (World Health Organization, 2015). It highlighted, in particular, the contribution of SSBs to intake of free sugars, and thus of excessive energy intake (World Health Organization, 2015). However, there is intense debate about how to achieve this (Frieden, 2010), with the beverage industry favouring individual approaches, such as education and provision of information, while the public health community supports structural approaches, such as those directed at price, availability and marketing (Jenkin et al., 2011, Mello et al., 2008, Popkin and Hawkes, 2015).

This debate should be informed by evidence. Why is the growth of sugar-sweetened beverages so much greater in some places than in others? Both supply and demand are likely to play a role. For example, the geographical spread of manufacturing or bottling/canning plants which, when coupled with improvements in transport infrastructure and urbanisation, have greatly increased the availability and affordability of consumer products, including SSBs, in many low and middle-income countries (Hawkes, 2005, Hills and Welford, 2005, Igumbor et al., 2012). Economic development, with rising disposable incomes, coupled with marketing campaigns that encourage aspiration to “western” lifestyles, increase demand for products bearing aspirations to global brand names. (Cassim, 2010, Popkin, 1999, Stuckler et al., 2011).

Recently, scholars have voiced concerns that trade and investment agreements, and the resulting market integration, facilitate the spread of sugar-sweetened beverages (Friel et al., 2013a, Friel et al., 2013b; ; Hawkes, 2005, Moodie et al., 2013, Stuckler et al., 2012, Stuckler and Siegel, 2011; A. M. Thow, 2009). A systematic review by Friel and colleagues found robust evidence that liberalisation of trade and foreign investment had been linked to changes in the food environments and diets, and specifically increased availability, accessibility, affordability, desirability, and consumption of food and sugary drinks linked to obesity and diet-related NCDs (Friel et al., 2013a, Friel et al., 2013b).

Yet, much of the existing scholarship on trade liberalisation and sugar-sweetened beverages tends to be qualitative and draw on case-study methodologies. For example, two case studies researching trade liberalisation policies in Central America, including the Central American-USA Free Trade Agreement, showed that lower tariffs and less restrictive non-tariff barriers (NTBs) had increased imports and overall availability of foodstuffs implicated in the nutrition transition (Hawkes and Thow, 2008; A. M. Thow and Hawkes, 2009). Similar studies have examined the North American Free Trade Agreement (Clark et al., 2012) and trade agreements involving Pacific islands (Hughes and Lawrence, 2005, Snowdon and Thow, 2013; A. M. Thow et al., 2011) and Ghana (A. M. Thow et al., 2014), among others. These case studies yield important insights into the potential mechanisms involved, but may not be generalizable to different national contexts.

There are relatively few quantitative studies of health impacts of trade integration. A systematic literature review of quantitative studies by Burns et al. showed an overall beneficial association between international trade or FDI and population health, but this review only addressed non-nutritional health outcomes (Burns et al., 2016). One cross-country longitudinal study examined the link between trade and investment liberalisation and sales of SSBs in LMICs (Stuckler et al., 2012). It found that higher levels of foreign direct investment inflows were associated with higher sales of unhealthy commodities, which included tobacco, alcohol, and processed foods and drinks. The study also found that LMICs entering into free trade agreements (FTAs) with the United States had 63.4% higher sales of soft drinks compared to those with similar levels of GDP and urbanisation that did not. Another recent study used a natural experimental design comparing Vietnam and the Philippines, showing that Vietnam's removal of restrictions on FDI following its accession to the World Trade Organisation (WTO) was associated with an increase in sales of SSBs not seen in the Philippines, which had joined the WTO some time previously (Schram et al., 2015). Most of this growth in sales of SSBs benefited multinational beverage companies, which gained access to the market after lifting of investment barriers, to the disadvantage of local companies. This latter point is important; opening of markets to multinational tobacco companies with their highly effective marketing techniques and global brands, for example through privatisation of former monopolies, is typically associated with increased cigarette sales (Gilmore et al., 2011).

These earlier studies made important contributions but had a number of limitations. One was the inability to differentiate imports from domestic consumption, and thus assess the extent to which greater consumption was driven by imports. Another was the inability to study factors that might mitigate the effects of opening of markets, such as the maintenance of tariff barriers designed to protect domestic manufacture. Tariffs are extremely controversial, but where the products traded are associated with adverse health effects, they could play a role by using price signals to counter the power of marketing by global producers. Unsurprisingly, those favouring free trade, including in substances hazardous to health, have sought to reduce or eliminate tariffs. However, given that there is at least a theoretical argument that they may act to protect health, in this case by reducing the growth in consumption of SSBs in LMICs, it is of interest to ask whether this is borne out by the evidence.

Here, to our knowledge for the first time, we combine data on SSB imports, tariffs, and SSBs sales to test these hypotheses across 44 LMICs. We use these data to ask whether higher tariffs attenuated imports of SSBs and whether increased SSBs imports per capita are associated with greater sales of SSBs.

## 2 Methods

### 2.1 Sources of data

We collected data on SSBs sales in retail and food service outlets over the period 2001 to 2014 from 44 LMICs using data from Euromonitor International, 2015 edition (Euromonitor International, 2015). Euromonitor provides market data based on private industry records for a total of 80 countries, of which 44 are LMICs during the studied period. It bases its estimates on multiple sources including information from official statistics, trade associations, the trade press, trade interviews, and its own estimates. Euromonitor is a harmonised source of data across countries. We note, however, that though it is very widely used, it is a proprietary product and, to our knowledge, has not been subject to independent evaluation of data quality. Sales of SSBs are measured in litres per capita and include carbonates, concentrates, juice, ready-to-drink coffees and teas, sports and energy drinks, and Asian specialty drinks, which include Bandung (rose syrup with milk), bird's nest, tamarind juice, ginger, lemongrass, jelly drinks, and drinks containing a limited amount of yogurt, among others. One limitation of these data as a measure of consumption is that they do not account for wastage. However, they overcome bias in self-reported consumption data, which tend to underestimate quantity consumed, and also have the benefit of comparability across countries, which is especially important in LMICs where epidemiological surveillance systems are often weak.

Sector-specific data on SSB imports for the period 2001–2014 were acquired from TradeMap, derived from the United Nations Commission on Trade and Development (UN COMTRADE) statistics (International Trade Centre, 2016). The data were compiled at the 4-digit level of the Harmonised Commodity Description and Definitions System (HS), which is an internationally standardised system to classify traded products. Imports of SSBs include products in two tariff lines: line 2009, which includes fruit juices and vegetable juices, unfermented and not containing added spirit, whether or not containing added sugar or other sweetening matter, and line 2202, which includes waters, including mineral waters and aerated waters, containing added sugar or other sweetening matter or flavoured, and other non-alcoholic beverages. All import data were in US$, adjusted for exchange rates and inflation. We used the inflation data provided by Euromonitor, which are based on the Consumer Price Index (CPI), to convert import data into real terms using 2001 as the base year. Import data were converted in imports per capita by dividing total imports by total population. Data on total population was obtained from Euromonitor, which relies on national statistics and UN data.

We evaluated trade liberalisation using data on tariffs of SSBs. Data on applied tariffs to the Most Favoured Nations, which are non-discriminatory tariffs charged on imports of WTO member countries, were compiled from the World Trade Organisation tariffs database (World Trade Organization, 2015). The data were compiled at the 4-digit level of the HS for the tariff lines 2202 and 2009. We computed the average of both tariff lines for the analyses. A higher tariff value indicates that the duties or taxes applied to imports are higher. Data were unavailable for seven out of the 44 countries, thus these were dropped from analyses including tariffs on SSBs imports.

Investment liberalisation was measured using data on foreign direct investment inflows as percentage of GDP. These data were obtained from Euromonitor, which sources its FDI data from the United Nations Conference on Trade And Development (UNCTAD).

To adjust for potential confounding in all models, we controlled for economic development – defined using GDP per capita – and level of urbanisation – defined using urban population as a percentage of total population – for the reasons noted in the introduction. Data on GDP per capita, adjusted for purchasing power parity (PPP) for comparability between countries, were taken from Euromonitor. Data on urban population as a percentage of total population were from the WDI Word Bank database.

### 2.2 Statistical models

Our econometric models included fixed effects to account for country-level characteristics that could influence the growth of SSBs. This is more conservative but preferable as between-country variations might be confounded by country-specific time-invariant unobserved factors correlated with the spread of SSBs. First, to investigate our first hypothesis, we examine the association of SSBs imports with tariff protections, controlling for potential confounders, as follows:

$$\text{ SSBs}\;{\text{Imports}}_{\text{it}}={\text{β}}_{0}+{\text{β}}_{1}SSBs\;Tariffs_{\text{it}}+{\text{β}}_{3}GDP_{\text{it}}+{\text{β}}_{4}Urbanisation+{\text{ν}}_{\text{i}}+{\text{ε}}_{\text{it}}$$

Second, to investigate our second hypothesis, we examine the association of SSBs imports with sales of SSBs, controlling for potential confounders:

$$\text{SSBs}\;{\text{Sales}}_{\text{it}}={\text{β}}_{0}+{\text{β}}_{1}SSBs\;Imports_{\text{it}}+{\text{β}}_{2}FDI_{\text{it}}+{\text{β}}_{3}GDP_{\text{it}}+{\text{β}}_{4}Urbanisation+{\text{ν}}_{\text{i}}+{\text{ε}}_{\text{it}}$$

Third, we examine the association of tariff protections on SSBs imports with sales of SSBs, as follows:

$$\text{SSBs}\;{\text{Sales}}_{\text{it}}={\text{β}}_{0}+{\text{β}}_{1}SSBs\;Tariffs_{\text{it}}+{\text{β}}_{2}FDI_{\text{it}}+{\text{β}}_{3}GDP_{\text{it}}+{\text{β}}_{4}Urbanisation+{\text{ν}}_{\text{i}}+{\text{ε}}_{\text{it}}$$

where *i* is the country and *t* is the year; SSBs Imports is imports per capita of SSBs in US$; to adjust for positive skew we logged these import data. Tariffs is applied tariffs on SSBs; GDP is GDP per capita adjusted for purchasing power parity; Urbanisation is urban population as a percentage of the total population; ν_i_ is an error term denoting country fixed effects and ε_it_ is an identically distributed random error. In subsequent models we evaluated the impact of SSBs imports on sales of SSBs, again using a fixed effects modelling framework. Robust clustered standard errors were used to reflect non-independence of country sampling. All statistical analyses were conducted using Stata 13.0.

## 3 Results

Table A1 in the Web Appendix presents average country-level descriptive statistics for the period 2001–2014, including GDP per capita in PPP, urban population as percentage of total population, Gini index, FDI inflows as percentage of GDP, SSBs sales in litres, SSBs imports in USD, SSBs tariffs, and diabetes prevalence rate. In addition, Figure A1 presents the yearly average imports of SSBs in USD for the period 2001–2014 and shows that there has been a substantial increase in imports of sugary drinks overtime in these LMICs. Figure A2 presents the average SSBs tariffs over the same period of time, where a lower tariff value indicates that taxes or duties applied to imports are lower. This figure shows that between 2001 and 2014 SSBs tariff levels have on average been reduced. Thus, the data presented in this latter figure is consistent with international efforts over this period of time to liberalise markets in developing countries through reducing or eliminating tariff and non-tariff barriers.

### 3.1 The impact of tariffs on imports

Table 1 shows the impact of tariffs of SSBs on imports of SSBs (Table 2). We observed that a one percent increase in tariffs was associated with a decrease of SSB imports by 5% (95% CI: 2.8%–7.3%). After adjusting for GDP per capita and percentage of urban population, this coefficient remained inversely and significantly associated with SSB imports (β: 2.9%; 95% CI: 0.9%–5%).

### 3.2 The impact of imports on sales

Table 2 then looks at how these changes in imports relate to sales of SSBs, derived from our cross-national statistical model. We observed that for every ten percent increase in SSB imports per capita, sales of SSBs increased by 0.96 L per person (95% CI: 0.64–1.28). After adjusting for FDI, GDP, and urbanisation, the coefficient for SSB imports was attenuated to 0.36 L, but remained significantly related to sales of SSBs (95% CI: 0.08–0.65). We observed that for every 1% increase in FDI as a percent of GDP, sales of SSBs increased by 0.34 L (95% CI: 0.12–0.56).

Consistent with previous findings, we found an association of greater GDP with higher sales of SSBs. Each US$100 increase in GDP per capita was associated with sales of an additional 0.22 L of SSBs per capita (95% CI: 0.13–0.32). We also observed that, in this case, urbanisation had no effect on SSB sales. Although superficially surprising, this is consistent with earlier observations that markets for SSBs in urban and rural areas of LMICs are now becoming saturated (Stuckler et al., 2012).

### 3.3 The impact of tariffs on sales

Table 3 looks at the association between tariffs of SSBs and sales of SSBs. Although we found that tariffs were inversely associated with imports, we did not observe a significant association between them (β: −0.47; 95% CI: −0.95–0.01), and the size of the coefficient was substantially reduced after adjusting for potentially confounding factors (β: −0.01; 95% CI: −0.28–0.27).

The average annual rise in SSB imports per capita was 17.9%, which, based on the results of the econometric model 2 of Table 2, is estimated to be equivalent to an annual increase of 0.65 L per person in our sample of LMICs. Cumulatively, over the 14 years period between 2001 and 2014, this translates into an increase in sales of 9.1 L per person. At the same time, sales of SSBs rose, on average, by 21.9 L per person. Thus, imports contributed about 40% of the observed rise in SSB sales.

### 3.4 Robustness checks

We conducted a series of robustness checks, testing our sample for outliers and model specification. Although fixed effects estimators are preferred to correct for country-specific conditions that could influence the spread of SSBs, we applied a test of overidentifying restrictions for panel data based on the Sargan-Hansen statistic, which statistically compares a fixed to a random effects model (Schaffer and Stillman, 2006 Oct). These results confirm the need for more conservative fixed effects estimates.

Next, because four countries, Chile, Latvia, Lithuania, and Uruguay, were no longer classified as LMICs in 2014, we re-ran our models excluding observations for these four countries in 2014. We did not find substantial differences in the results. In addition, we included a linear time trend in our models and found that our analyses were unchanged.

Lastly, we performed additional analyses using an alternative measure of trade integration, the Index of Globalization, created by Dreher et al. and published by ETH Zurich (Dreher, 2006, Dreher et al., 2008) (see Web Appendix Box A1), differentiating indicators of economic, social, and political globalization. Using these indices in our model rather than SSB imports, we found that every one percent increase in economic globalisation was positively and significantly associated with sales of SSBs (*β* = 0.33; 95% CI: 0.03–0.64). Neither social globalisation nor political globalisation had a significant association with SSB sales. These analyses are presented in the Web Appendix.

## 4 Discussion

Our results yield two main findings. First, we observed that reduction in tariffs was associated with greater imports of SSBs to LMICs. Second, we observed a strong association between SSB imports and overall sales of SSBs. Our estimates indicate that about 40% of the observed rise in SSB sales over the past 14 years in LMICs could be accounted for by additional imports. Obviously, these findings, on their own, do not indicate causation. Imports and sales may be linked bi-directionally to each other through supply and demand, with each influencing the other in ways that cannot be discerned precisely with these data. However, while it is theoretically possible that greater imports might strengthen domestic political pressure for tariff reduction, in this case the association is very much more likely to flow from tariff reduction, typically as part of wider discussions on trade, to imports and sales of SSBs (Kreinin, 1961, Wang, 2001).

Given that tariffs affect SSBs imports, which, in turn, affect SSBs sales, we believe that the non-significant direct link between tariffs and SSBs sales might be due to lack of statistical power to capture the effect of tariffs. The direct effect size of tariffs on sales might be relatively small as to be captured in our model, as its effect on the SSBs domestic markets occurs necessarily through import levels, which are additionally influenced by factors in addition to tariffs, which could include, for example, demand, local productivity, quality, and marketing.

The simultaneous effect of international trade and foreign investment on SSBs sales points out multiple pathways within market liberalisation that lead to increased sales of SSBs. Previous research has emphasised the importance of FDI flowing from food and beverage multinational companies based in high-income countries to markets of LMICs in promoting local production and consumption of SSBs (Baker et al., 2016, Clark et al., 2012, Hawkes, 2005, Schram et al., 2015, Schram et al., 2013, Stuckler et al., 2012). The data in Figure A1 shows that at the same time there was also a substantial increase in imports of SSBs into LMICs. Imports of SSBs might be especially relevant in affecting local markets through regional trade and in small insular countries or in countries with growing SSBs markets that still lack a well-developed infrastructure for the production of SSBs. For instance, in Southeast Asia, Malaysia and Vietnam had a high level of SSBs imports from Thailand, which was one of the main exporters of SSBs to these countries and might be acting as a regional hub (International Trade Centre, 2016). In Latin America, Bolivia mostly imported SSBs from neighbouring countries Peru and Argentina, while the latter had high imports from the United States, Austria, Switzerland, and Brazil (International Trade Centre, 2016). Some insular countries, such as Fiji, Samoa, Nauru, and Cook Islands, impose higher tariffs in SSBs and other sugary foods as an economic tool to regulate food environments through food affordability and purchase incentives (World Cancer Research Fund International, 2016), which points that imports of SSBs might be a pathway significantly affecting the availability of sugary drinks in these markets. Nonetheless, the simultaneous impact of investment and trade liberalisation in food environments of LMICs deserves further investigation.

There are several directions for future research. Trade policies and foreign direct investment could impact on the availability of SSB in a country by increasing the supply of foodstuffs required for manufacturing of SSBs, increasing domestic manufacturing facilities. Thus, future work should explore how trade liberalisation impacts on ingredients used for the manufacturing of SSBs, such as high fructose corn syrup, and in turn, on the supply of SSBs produced within countries. These types of analyses could also be extended to explore their impact on population health outcomes, such as diabetes, obesity, and oral health.

As with all cross-national statistical studies, our analysis has several limitations. First, we were unable to obtain sector-specific FDI data. This would have added greater analytic specificity to our models and enabled us to build upon previous work showing a positive relationship between FDI and sales of SSB (Stuckler et al., 2012). We also included total FDI in our models, but this could have biased our estimates toward the null if there is variation in the extent to which total FDI is associated with FDI by SSB producers. Second, again due to lack of data availability, most of the countries in the sample are middle-income countries, with only four southern African countries included in the sample. Nonetheless, we would expect our findings to be generalizable to these contexts, as a recent case-study of southern African countries highlighted how trade and investment liberalisation was linked to increasing availability of SSBs (A. M. Thow et al., 2015a, Thow et al., 2015b). Third, another important data limitation in our analyses is that Euromonitor data excludes bottled water whereas the import data does contain bottled water, which might bias our results toward the null. Fourth, while we used models that controlled for country fixed-effects, there may be unobserved time-varying factors that influenced the sales of SSBs in these countries over this period. The introduction of a soda tax in Mexico is an example of what can happen (Colchero et al., 2016). Fifth, this study could not examine the effect of non-tariff barriers. Some, such as restrictions on sales outlets or marketing, are unlikely in LMICs but one possibility is the presence of a strong domestic producer that dominates the market. However, as non-tariff barriers also act in ways that resemble the effects of tariffs, because the purpose of both is to impose restrictions on imports, we would expect them to have a similar effect on trade as tariffs. Therefore, as we are not examining the effect of non-tariff barriers, our results could be an overestimation of the effect of tariffs. Future research will benefit from exploring the impact of both tariffs and non-tariff barriers on the food environments. Sixth, changes in tariffs, and by extension availability and prices, could differentially affect socio-economic groups. With the aggregate data available to us we cannot answer this question. Our results could be both overestimating and underestimating the observed effects in some subpopulations. However, a new systematic review finds that the impact of price rises across socio-economic groups is essentially constant, and while tax rises are regressive, this is only to a very small extent (Backholer et al., 2016). There is now good evidence supporting a link between taxes and sales (Alagiyawanna et al., 2015, Backholer et al., 2016, Briggs et al., 2013, Colchero et al., 2016, Falbe et al., 2016). A recent study on the imposition of price increases on SSBs via taxes in Berkeley shows that the price elasticity was much larger than expected (Falbe et al., 2016). This suggests that it may be possible to reap the benefits of trade liberalisation (Burns et al., 2016, Chemingui et al., 2010, Umaña-Peña et al., 2014) while countering the problems through targeted taxes, although clearly it will be necessary to ensure that these are non-discriminatory to comply with trade deals.

Our results have important policy implications for a global environment characterised, at present, by an ever-greater consumption of SSBs, especially in LMICs, as well as an increasing number of regional and global agreements seeking further reductions in trade barriers. Given growing evidence of public health concerns associated with trade and investment liberalisation (Baker et al., 2016, Lencucha et al., 2016, McNamara, 2015, Schram et al., 2015; A. M. Thow et al., 2015a, Thow et al., 2015b; Anne Marie Thow et al., 2015a, Thow et al., 2015b; A. M. T. Thow et al., 2015c), concerns borne out by the results in this study, advocacy groups should demand that public health should be prioritised in drafting of trade agreements. These results indicate that tariffs can have a role in counteracting the entrance of harmful products in emerging markets of LMICs and therefore could be an effective policy tool to regulate food environments by discouraging the availability and affordability of unhealthy products. Some countries such as Fiji, Samoa, Nauru, French Polynesia, and Cook Islands, impose taxes on imports of SSBs (World Cancer Research Fund International, 2016), highlighting how such policies are feasible. In light of the rapid growth of consumption of SSBs and clear evidence linking consumption to worse health, there is an urgent need for more countries to align trade and investment priorities with public health.

## Conflicts of interest

None to declare.

## Figures and Tables

**Table 1:** Impact of SSBs tariffs on log imports of SSBs in US$ per capita – 37 LMIC, 2001–2014.

	Log imports per capita of SSBs in US$	
	(1)	(2)
Per one point increase in SSBs tariffs	−0.050^∗∗^ (0.011)	−0.029^∗∗^ (0.010)
Per1% increase in FDI as % of GDP		0.024 (0.019)
Per US$100 increase in GDP per capita, PPP		0.011^∗^ (0.0044)
Per 1% increase in urban population		0.030 (0.037)
Linear time trend		
Number of country-years	385	385
*R*^2^	0.157	0.410

Constant included in models but not shown.

All models include country fixed effects and report clustered standard errors in parentheses.

^∗^*p* < 0.05, ^∗∗^*p* < 0.01.

**Table 2:** Impact of imports of SSBs on per capita sales of SSBs – 44 LMICs, 2001–2014.

	Per capita sales of SSBs (litres)	
	(1)	(2)
Per 10% increase in SSBs imports per capita in USD	0.96^∗∗^ (0.16)	0.364^∗^ (0.141)
Per1% increase in FDI as % of GDP		0.34^∗∗^ (0.11)
Per US$100 increase in GDP per capita, PPP		0.22^∗∗^ (0.046)
Per 1% increase in urban population		0.22 (0.30)
Number of country-years	581	581
*R*^2^	0.346	0.599

Constant included in models but not shown.

All models include country fixed effects and report clustered standard errors in parentheses.

^∗^*p* < 0.05, ^∗∗^*p* < 0.01.

**Table 3:** Impact of SSBs tariffs on per capita sales of SSBs – 37 LMICs, 2001–2014.

	Per capita sales of SSBs (litres)	
	(1)	(2)
Per one point increase in SSBs tariffs	−0.47 (0.24)	−0.0097 (0.14)
Per1% increase in FDI as % of GDP		0.26 (0.33)
Per US$100 increase in GDP per capita, PPP		0.34^∗∗^ (0.11)
Per 1% increase in urban population		0.049 (0.67)
Number of country-years	425	425
*R*^2^	0.046	0.527

Constant included in models but not shown.

All models include country fixed effects and report clustered standard errors in parentheses.

^∗^*p* < 0.05, ^∗∗^*p* < 0.01.
