# Supplementary material for: Is trade liberalisation a vector for the spread of sugar-sweetened beverages? A cross-national longitudinal analysis of 44 low- and middle-income countries
Source: Soc Sci Med. 2017 Jan;172:21–7. doi: 10.1016/j.socscimed.2016.11.001 (PMC5224188; doi:10.1016/j.socscimed.2016.11.001)
Supplement: Supplementary file 1 [file mmc1.docx]

**Electronic Supplementary Material**

Figure A1 – Average imports of SSBs in US dollars, 44 LMICs, 2001–2014.

Figure A2 – Average SSBs tariffs, 37 LMICs, 2001–2014.

Table A1 – Average country-level descriptive statistics, 44 LMICs, 2001-2014.

Table A2 - Impact of SSBs tariffs on log imports of SSBs in US$ per capita adjusted for linear time trends, 37 LMIC, 2001–2014.

Table A3 - Impact of imports of SSBs on per capita sales of SSBs adjusted for linear time trends, 44 LMICs, 2001–2014.

Table A4 - Impact of SSBs tariffs on per capita sales of SSBs adjusted for linear time trends, 37 LMICs, 2001–2014.

Box A1 - Index of Globalisation.

Table A5 - Impact of globalisation measures on per capita sales of SSBs – 43 LMICs, 2000–2012.

References

Figure A1 – Average imports of SSBs in US dollars, 44 LMICs, 2001–2014.


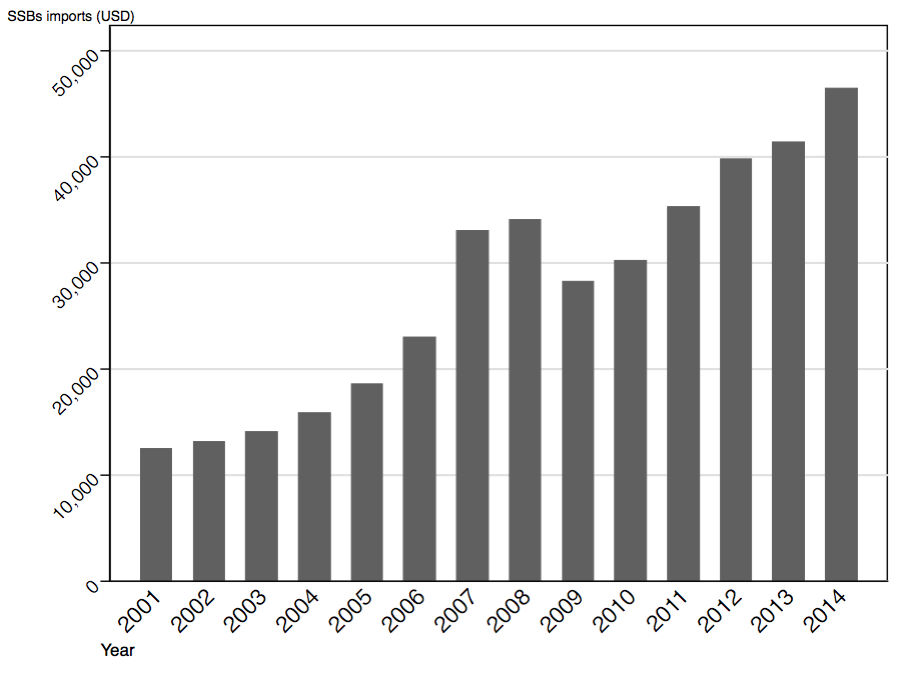


Figure A2 – Average SSBs tariffs, 37 LMICs, 2001–2014.


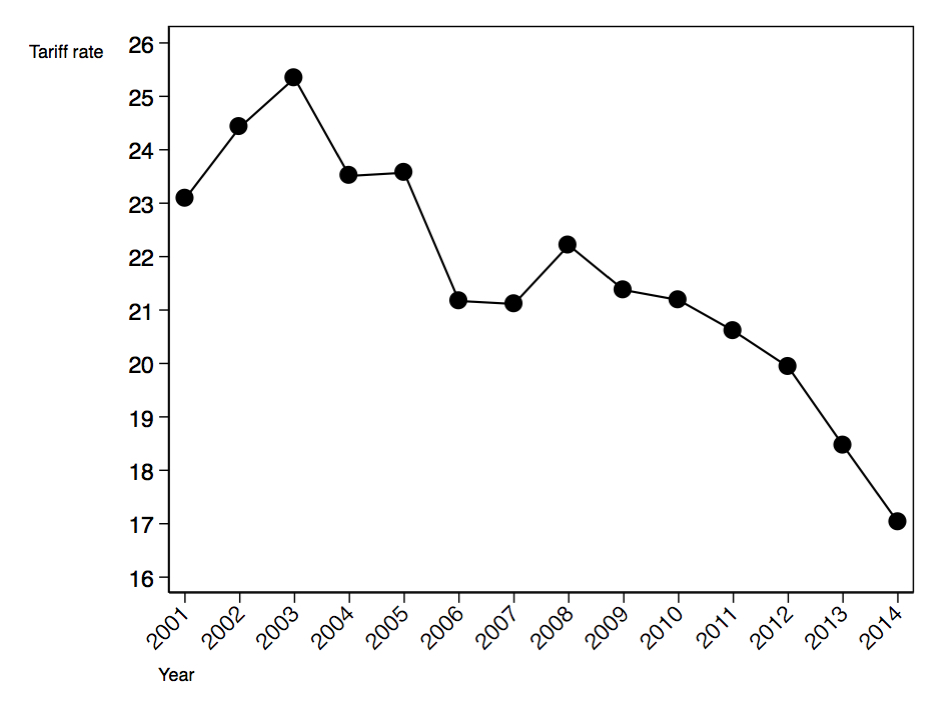


Table A1 – Average country-level descriptive statistics, 44 LMICs, 2001-2014.

| Country | **GDP per capita in USD, PPP** | **Gini index** | **Urban population (as % of total population)** | **FDI inflows (as % of GDP)** | **SSBs sales (litres)** | **SSBs imports (USD)** | **SSBs applied tariff rates** | **Diabetes prevalence rate** |
| --- | --- | --- | --- | --- | --- | --- | --- | --- |
| Algeria | 11806.6 | 36.8 | 65.6 | 1.6 | 28.7 | 13800.4 |  | 4.7 |
| Argentina | 16758.7 | 43.0 | 90.5 | 1.8 | 171.4 | 9461.4 | 17.4 | 6.1 |
| Azerbaijan | 11214.1 | 39.1 | 52.9 | 8.9 | 25.7 | 10270.4 |  | 4.1 |
| Belarus | 12467.7 | 32.4 | 73.5 | 2.5 | 46.9 | 21651.9 | 5.0 | 7.9 |
| Bolivia | 4852.2 | 53.8 | 65.3 | 3.4 | 63.0 | 4998.3 | 12.3 | 5.8 |
| Bosnia-Herzegovina | 7980.5 | 36.7 | 39.3 | 3.8 | 44.7 | 34827.0 |  | 9.4 |
| Brazil | 12729.1 | 52.3 | 83.6 | 2.5 | 82.2 | 27942.4 | 17.3 | 7.3 |
| Bulgaria | 13563.8 | 35.8 | 71.4 | 10.0 | 87.4 | 30214.4 | 33.2 | 7.7 |
| Cameroon | 2529.2 | 47.2 | 50.0 | 1.8 | 11.5 | 4314.1 | 29.9 | 3.5 |
| Chile | 17200.9 | 40.1 | 88.0 | 6.7 | 141.3 | 31105.6 | 6.2 | 8.4 |
| China | 7342.0 | 47.1 | 45.8 | 2.4 | 21.8 | 151755.8 | 25.6 | 6.9 |
| Colombia | 9688.3 | 55.5 | 74.3 | 3.7 | 71.4 | 15363.1 | 18.0 | 5.8 |
| Costa Rica | 11285.1 | 49.5 | 68.6 | 4.9 | 102.4 | 17391.8 | 13.2 | 5.5 |
| Dominican Republic | 9942.6 | 49.6 | 70.4 | 3.8 | 78.4 | 15410.9 | 19.3 | 8.7 |
| Ecuador | 8832.6 | 53.0 | 62.2 | 1.3 | 69.5 | 13863.7 | 23.7 | 5.5 |
| Egypt | 9048.2 | 36.0 | 43.0 | 3.3 | 24.7 | 14888.4 | 30.4 | 12.6 |
| Georgia | 5157.7 | 46.4 | 52.7 | 8.4 | 28.7 | 6802.9 | 15.2 | 4.3 |
| Guatemala | 6325.1 | 56.4 | 48.3 | 1.9 | 95.3 | 38230.2 | 13.3 | 6.7 |
| India | 3789.7 | 38.6 | 30.1 | 1.7 | 2.7 | 33514.2 | 30.6 | 7.8 |
| Indonesia | 7341.6 | 36.4 | 47.9 | 1.3 | 11.0 | 33202.2 | 6.8 | 6.0 |
| Iran | 14669.9 | 44.8 | 69.0 | 1.1 | 40.6 | 8047.0 |  | 7.6 |
| Kazakhstan | 16892.1 | 30.5 | 54.3 | 8.2 | 47.1 | 38587.4 |  | 4.9 |
| Kenya | 2256.4 | 49.8 | 22.6 | 0.6 | 8.9 | 3715.8 | 26.3 | 2.8 |
| Latvia | 17547.5 | 35.8 | 67.8 | 3.6 | 53.5 | 29468.3 | 10.1 | 5.7 |
| Lithuania | 19033.9 | 36.4 | 66.7 | 2.9 | 44.3 | 47913.3 | 12.0 | 4.8 |
| Macedonia | 10190.3 | 40.0 | 57.4 | 3.3 | 71.8 | 14995.7 | 27.6 | 8.9 |
| Malaysia | 18690.3 | 44.4 | 68.7 | 3.0 | 32.3 | 60218.8 | 12.7 | 9.0 |
| Mexico | 15011.7 | 50.4 | 77.1 | 2.7 | 169.4 | 114191.3 | 20.7 | 8.9 |
| Morocco | 5868.4 | 41.0 | 56.5 | 3.0 | 22.0 | 14609.7 | 46.8 | 5.6 |
| Nigeria | 4181.4 | 53.1 | 41.3 | 2.1 | 11.2 | 26664.6 | 28.4 | 4.2 |
| Pakistan | 3460.7 | 36.7 | 35.7 | 1.5 | 6.1 | 6236.0 | 29.2 | 7.2 |
| Peru | 8400.1 | 48.6 | 75.9 | 4.2 | 59.0 | 14921.4 | 12.7 | 4.7 |
| Philippines | 4971.1 | 46.9 | 46.0 | 1.3 | 42.3 | 23573.9 | 7.9 | 6.4 |
| Romania | 14779.2 | 32.9 | 53.5 | 4.0 | 67.2 | 33651.2 |  | 5.8 |
| Serbia | 10665.8 | 34.1 | 54.8 | 6.3 | 85.1 | 12857.0 | 24.3 | 12.3 |
| South Africa | 10773.9 | 63.6 | 60.9 | 1.8 | 78.5 | 59361.6 | 20.9 | 6.4 |
| Thailand | 11058.6 | 46.1 | 40.8 | 3.1 | 47.7 | 35492.2 | 44.5 | 5.4 |
| Tunisia | 8972.8 | 39.5 | 65.4 | 3.6 | 47.1 | 5564.7 | 47.5 | 7.5 |
| Turkey | 14955.7 | 39.9 | 69.2 | 1.8 | 48.9 | 17279.7 | 34.6 | 12.0 |
| Ukraine | 7224.6 | 34.1 | 68.3 | 4.1 | 40.6 | 44352.8 | 7.3 | 3.5 |
| Uruguay | 14052.8 | 42.1 | 93.8 | 4.6 | 95.4 | 4636.2 | 17.4 | 6.6 |
| Uzbekistan | 3615.3 | 39.3 | 36.5 | 1.8 | 13.7 | 673.7 |  | 4.5 |
| Venezuela | 15004.7 | 43.1 | 88.7 | 1.2 | 90.3 | 8762.4 | 19.2 | 5.8 |
| Vietnam | 3766.4 | 42.9 | 28.9 | 5.4 | 14.0 | 8497.8 | 40.9 | 4.0 |

Table A2 - Impact of SSBs tariffs on log imports of SSBs in US$ per capita adjusted for linear time trends, 37 LMIC, 2001–2014.

|  | Log imports per capita of SSBs in US$ | | |
| --- | --- | --- | --- |
|  | (1) | (2) | (3) |
|  |  |  |  |
| Per one point increase in SSBs tariffs | -0.050^**^ | -0.029^**^ | -0.024^*^ |
|  | (0.011) | (0.010) | (0.0098) |
|  |  |  |  |
| Per1% increase in FDI as % of GDP |  | 0.024 | 0.031 |
|  |  | (0.019) | (0.019) |
|  |  |  |  |
| Per US$100 increase in GDP per capita, PPP |  | 0.011^*^ | 0.0044 |
|  |  | (0.0044) | (0.0050) |
|  |  |  |  |
| Per 1% increase in urban population |  | 0.030 | 0.0085 |
|  |  | (0.037) | (0.038) |
|  |  |  |  |
| Linear time trend |  |  | 0.055 |
|  |  |  | (0.034) |
|  |  |  |  |
| Number of country-years | 385 | 385 | 385 |
| *R*^2^ | 0.157 | 0.410 | 0.434 |

Constant included in models but not shown

All models include country fixed effects and report clustered standard errors in parentheses.

^*^ *p* < 0.05, ^**^ *p* < 0.01

Table A3 - Impact of imports of SSBs on per capita sales of SSBs adjusted for linear time trends, 44 LMICs, 2001–2014.

|  | Per capita sales of SSBs (liters) | | |
| --- | --- | --- | --- |
|  | (1) | (2) | (3) |
|  |  |  |  |
| Per 10% increase in SSBs imports per capita in USD | 0.96^**^ | 0.364^*^ | 0.318^*^ |
|  | (0.16) | (0.141) | (0.135) |
|  |  |  |  |
| Per1% increase in FDI as % of GDP |  | 0.34^**^ | 0.33^**^ |
|  |  | (0.11) | (0.10) |
|  |  |  |  |
| Per US$100 increase in GDP per capita, PPP |  | 0.22^**^ | 0.17^**^ |
|  |  | (0.046) | (0.06) |
|  |  |  |  |
| Per 1% increase in urban population |  | 0.22 | -0.085 |
|  |  | (0.30) | (0.31) |
|  |  |  |  |
| Linear time trend |  |  | 0.60 |
|  |  |  | (0.34) |
|  |  |  |  |
| Number of country-years | 581 | 581 | 581 |
| *R*^2^ | 0.346 | 0.599 | 0.610 |

Constant included in models but not shown

All models include country fixed effects and report clustered standard errors in parentheses.

^*^ *p* < 0.05, ^**^ *p* < 0.01

Table A4 - Impact of SSBs tariffs on per capita sales of SSBs adjusted for linear time trends, 37 LMICs, 2001–2014.

|  | Per capita sales of SSBs (liters) | | |
| --- | --- | --- | --- |
|  | (1) | (2) | (3) |
|  |  |  |  |
| Per one point increase in SSBs tariffs | -0.47 | -0.0097 | 0.052 |
|  | (0.24) | (0.14) | (0.14) |
|  |  |  |  |
| Per1% increase in FDI as % of GDP |  | 0.26 | 0.34 |
|  |  | (0.33) | (0.32) |
|  |  |  |  |
| Per US$100 increase in GDP per capita, PPP |  | 0.34^**^ | 0.25 |
|  |  | (0.11) | (0.15) |
|  |  |  |  |
| Per 1% increase in urban population |  | 0.049 | -0.27 |
|  |  | (0.67) | (0.58) |
|  |  |  |  |
| Linear time trend |  |  | 0.70 |
|  |  |  | (0.58) |
|  |  |  |  |
| Number of country-years | 425 | 425 | 425 |
| *R*^2^ | 0.046 | 0.527 | 0.541 |

Constant included in models but not shown

All models include country fixed effects and report clustered standard errors in parentheses.

^*^ *p* < 0.05, ^**^ *p* < 0.01

Box A1 - Index of Globalisation.

| The KOF Index of Globalisation measures three main dimensions of globalisation, namely economic integration, social integration, and political integration (Dreher, 2006), in a scale from 0 to 100. Descriptive statistics of these three variables are provided in the text in table 1.  “More specifically, the three dimensions of the KOF index are defined as:   - *economic globalisation*, characterised as long distance flows of goods, capital, and services as well as information and perceptions that accompany market exchanges; - *political globalisation*, characterised by a diffusion of government policies; and - *social globalisation*, expressed as the spread of ideas, information, images, and people” (KOF Index of Globalization, 2016a)   Economic globalisation includes measures of actual flows and economic restrictions. Some examples of the variables included in the index economic globalisation are FDI, trade openness, portfolio investment, mean tariff rate, and hidden import barriers. The index political globalisation includes, for example, variables measuring the number of memberships that a country has in international organisation and the number of embassies in a country. The index social globalisation includes measures of data on personal contact, data on information flows, and data on cultural proximity. Some examples of the variables included in these sub-indices are telephone traffic, Internet users, and trade in books.  For an exhaustive list of the variables included in the index and their weights see (Dreher, 2006; Dreher, Gaston, & Martens, 2008; KOF Index of Globalization, 2016b). There is no data available for Uzbekistan; therefore the analyses using the Index of globalisation only include 43 countries for the period 2000–2012, the latest data available. |
| --- |

Table A5 - Impact of globalisation measures on per capita sales of SSBs – 43 LMICs, 2000–2012.

|  | Per capita sales of SSBs (litres) | | |
| --- | --- | --- | --- |
|  | (1) | (2) | (3) |
| Per 1 point increase in economic globalisation | 0.36^*^ | 0.33^*^ | 0.33^*^ |
|  | (0.14) | (0.14) | (0.15) |
|  |  |  |  |
| Per 1 point increase in social globalisation |  | 0.23 | 0.23 |
|  |  | (0.26) | (0.27) |
|  |  |  |  |
| Per 1 point increase in political globalisation |  |  | 0.0011 |
|  |  |  | (0.16) |
|  |  |  |  |
| Per US$100 increase in GDP per capita PPP | 0.28^**^ | 0.26^**^ | 0.26^**^ |
|  | (0.048) | (0.051) | (0.059) |
|  |  |  |  |
| Per 1% increase in urban population (% of total) | 0.13 | 0.061 | 0.061 |
|  | (0.34) | (0.35) | (0.35) |
|  |  |  |  |
| Number of country-years | 559 | 559 | 559 |
| *R*^2^ | 0.592 | 0.594 | 0.594 |

Constant included in model but not shown

All models include fixed country effects and report clustered standard errors in parentheses.

^*^ *p* < 0.05, ^**^ *p* < 0.01

References

Dreher, A. (2006). Does globalization affect growth? Evidence from a new index of globalization. *Applied Economics, 38*(10), 1091-1110. doi:10.1080/00036840500392078

Dreher, A., Gaston, N., & Martens, P. (2008). *Measuring Globalization - Gauging its Consequence*. New York: Springer.

KOF Index of Globalization. (2016a). Method of calculation. Retrieved from <http://globalization.kof.ethz.ch/media/filer_public/2016/03/03/method_2016.pdf>

KOF Index of Globalization. (2016b). Variables and weights. Retrieved from <http://globalization.kof.ethz.ch/media/filer_public/2016/03/03/variables_2016.pdf>
